# Practical sampling of constraint-based models: Optimized thinning boosts CHRR performance

**DOI:** 10.1371/journal.pcbi.1011378

**Published:** 2023-08-11

**Authors:** Johann F. Jadebeck, Wolfgang Wiechert, Katharina Nöh

**Affiliations:** 1 Institute of Bio- and Geosciences, IBG-1: Biotechnology, Forschungszentrum Jülich, Jülich, Germany; 2 Computational Systems Biotechnology (AVT.CSB), RWTH Aachen University, Aachen, Germany; CPERI, GREECE

## Abstract

Thinning is a sub-sampling technique to reduce the memory footprint of Markov chain Monte Carlo. Despite being commonly used, thinning is rarely considered efficient. For sampling constraint-based models, a highly relevant use-case in systems biology, we here demonstrate that thinning boosts computational and, thereby, sampling efficiencies of the widely used Coordinate Hit-and-Run with Rounding (CHRR) algorithm. By benchmarking CHRR with thinning with simplices and genome-scale metabolic networks of up to thousands of dimensions, we find a substantial increase in computational efficiency compared to unthinned CHRR, in our examples by orders of magnitude, as measured by the effective sample size per time (*ESS*/*t*), with performance gains growing with polytope (effective network) dimension. Using a set of benchmark models we derive a ready-to-apply guideline for tuning thinning to efficient and effective use of compute resources without requiring additional coding effort. Our guideline is validated using three (out-of-sample) large-scale networks and we show that it allows sampling convex polytopes uniformly to convergence in a fraction of time, thereby unlocking the rigorous investigation of hitherto intractable models. The derivation of our guideline is explained in detail, allowing future researchers to update it as needed as new model classes and more training data becomes available. CHRR with deliberate utilization of thinning thereby paves the way to keep pace with progressing model sizes derived with the constraint-based reconstruction and analysis (COBRA) tool set. Sampling and evaluation pipelines are available at https://jugit.fz-juelich.de/IBG-1/ModSim/fluxomics/chrrt.

## Author summary

Analyzing the parameter spaces of genome-scale metabolic models (GEM) by means of Markov chain Monte Carlo (MCMC) sampling has become a key method in systems biology. In this context, sub-sampling, or *thinning*, reduces storage requirements and post-processing efforts of the immense sample volumes. However, sub-sampling is typically applied without due consideration, despite statisticians arguing that, by increasing the variance of the resulting estimate, thinning is almost always statistically inefficient. Considering synthetic and real sampling problems of widely varying complexity, we show that for the state-of-the-art uniform MCMC sampling algorithm, Coordinate Hit-and-Run with Rounding (CHRR), thinning has dramatic consequences on sampling performances: Our benchmarks reveal that sub-sampling boosts CHRR efficiencies, often by orders of magnitude, and the performance gain scales with problem dimension. For the studied problem classes, we provide simple rules of thumb for performance-optimized thinning, for which we give out-of-sample evidence. Utilization of our thinning guideline for GEMs, hence, keeps promise to solve hitherto inaccessible sampling problems, while for large scale networks its application can make the difference between immediate sampling success and repeated failures. In summary, on the one hand, optimized tuning of CHRR is now easily possible. On the other hand, thinning choice has to be registered when benchmarking CHRR implementations with regard to their running times.

## 1 Introduction

Constraint-based modelling of metabolism provides a versatile mathematical framework for integrating genomic and biochemical knowledge with multi-omics data and to interrogate network models [1–3]. As such, the constraint-based reconstruction and analysis (COBRA) methodology has established an indispensable tool set with numerous applications in metabolic engineering, biotechnology, microbiology, and systems as well as synthetic biology [4–7]. Nowadays, genome-scale models (GEMs) are routinely reconstructed from genomic data using automatized workflows [8]. Powerful reconstruction pipelines such as CaveMe [9], AGORA2 [10], or metaGEM [11] generate high-quality models with increasing sizes and at accelerated speed [12]. For instance, recently these tools enabled to generate more than 7,300 context-specific yeast GEMs [10]. Multi-organismal GEM repositories are publicly available, such as the BiGG database [13, 14] that contains 108 models, with the largest metabolic model, *Recon3D* [15], accounting for more than ten thousand reactions. Nowadays, modeling initiatives are starting to target the description of microbial communities, pan- and meta-genomes, and even the whole human body [16–18], therewith escalating model sizes further.

GEMs are particularly useful to predict phenotypes, to suggest genetic interventions, e.g., by applying bi-level strain design approaches [19], and to foresee the outcome of experiments [20]. Besides comprehensiveness, the capabilities of GEMs to deliver useful insights is intimately tied to the knowledge of the unknown model parameters, i.e., the intra- and extracellular metabolic reaction rates (fluxes). Here, in particular, enzyme constraints have been shown to effectively improve the models’ prediction performances [21]. The stoichiometry-induced mass balances at steady-state and further constraints introduce linear dependencies among the fluxes, implying that all feasible flux configurations are located within a convex hyper-polytope [22, 23], with a dimension dictated by the nullspace of the stoichiometric matrix. Interestingly, such convex polytopes as solution spaces arise in many applied modeling contexts, including operations research [24], ecological modeling [25], computational finance [26], astronomy [27], physics [28], and everywhere where network-flow problems are to be solved.

The expansion in GEM sizes has raised the need for developing scalable tools to analyze the high-dimensional flux solution spaces. In this context, uniform convex polytope sampling (UCPS), i.e., drawing representative random numbers from a truncated uniform distribution defined over the bounded flux polytope, is routinely used for characterizing the solution spaces of metabolic [29, 30] and gene networks [31], identification of metabolic flux couplings [32], design of experiments [20, 33], and assessing the effects of uncertainties in biochemical network formulations [34, 35]. A simple UCPS strategy is *rejection sampling*, a Monte Carlo technique that iteratively generates samples within easily accessible objects such as polytope-enclosing (hyper)cubes, where it keeps only those draws that are located interior to the polytope, until the truncated uniform target distribution is approximately covered [36]. While initial efforts in the field of constraint-based modeling used this technique [37], rejection sampling was quickly recognized to be computationally infeasible for more comprehensive network models, because for every feasible sample the number of rejections grows super-exponentially with polytope (effective model) dimension.

The class of Hit-and-Run (HR) algorithms has been developed to overcome this bottleneck [38]. HR is a Markov chain Monte Carlo (MCMC) technique, which, starting from an initial flux configuration within the polytope, generates a sequence of flux states (a Markov chain) by drawing randomly from inner chords directed in random directions [39]. As a result, the generated states (or a fraction of them) are called *MCMC samples*. These samples, albeit being correlated by construction, approximate the target distribution asymptotically [40].

How quickly the target distribution is approximated well in terms of wall-clock time, the so-called *sampling efficiency*, is critical for practical applicability of HR. Sampling efficiency is determined by two components: (1) the statistical efficiency, which specifies how well a set of samples represents a target distribution, and (2) the computational efficiency of the HR sampling algorithm, i.e., how many computational operations are required per generated state. Formally, the statistical efficiency of *N* samples produced by a Markov chain is given by the *effective sample size* (*ESS*) [36]:

$$\begin{array}{c} ESS=\frac{N}{1+2\cdot \sum_{t=1}^{\infty }\rho_{t}} \end{array}$$ (1)

with *ρ*_*t*_ being the autocorrelation of the sequence at lag *t*. The sampling efficiency of the MCMC implementation is then measured by the *ESS* normalized to wall clock time *t*, i.e., *ESS*/*t*.

According to Eq (1), two strategies to raise MCMC sampling efficiencies are (1) reducing the computational cost per sample, and (2) decreasing the autocorrelation between subsequent states. For HR, the cost per sample is reduced by updating the states in a pure coordinate-wise fashion instead of drawing random directions, a technique also known as Gibbs sampling [41]. To decrease the autocorrelation, the average distance between subsequent states is to be increased. In UCPS, the achievable distance is limited by the geometry of the polytope. Intuitively, in isotropic geometries on average longer steps are possible than in anisotropic spaces. Since heterogeneous parameter scales in GEMs are the rule, an affine transformation is employed to homogenize the flux scales prior to sampling, the so-called *rounding transformation* [22]. After sampling, the states have to be mapped back to the original space by inverting the rounding transformation. Together, the two strategies culminated in the celebrated Coordinate Hit-and-Run with Rounding (CHRR) algorithm [42], the tried-and-tested workhorse for UCPS in the COBRA domain [4, 43, 44], with several implementations being available [42, 45–47].

CHRR is commonly applied together with *thinning*, a MCMC post-processing technique, to reduce the memory footprint of the sampling results. The simplest and most widely used form of thinning is *fixed frequency* thinning with thinning constant *τ*, which means that only every *τ*^th^ sample is kept. While thinning indeed reduces the autocorrelation of the samples generated by CHRR, the reduction comes at the cost of producing an approximation of the target distribution that is less accurate. Indeed, thinning is known to be statistically inefficient in all but very few cases [48, 49].

**Fig 1:**
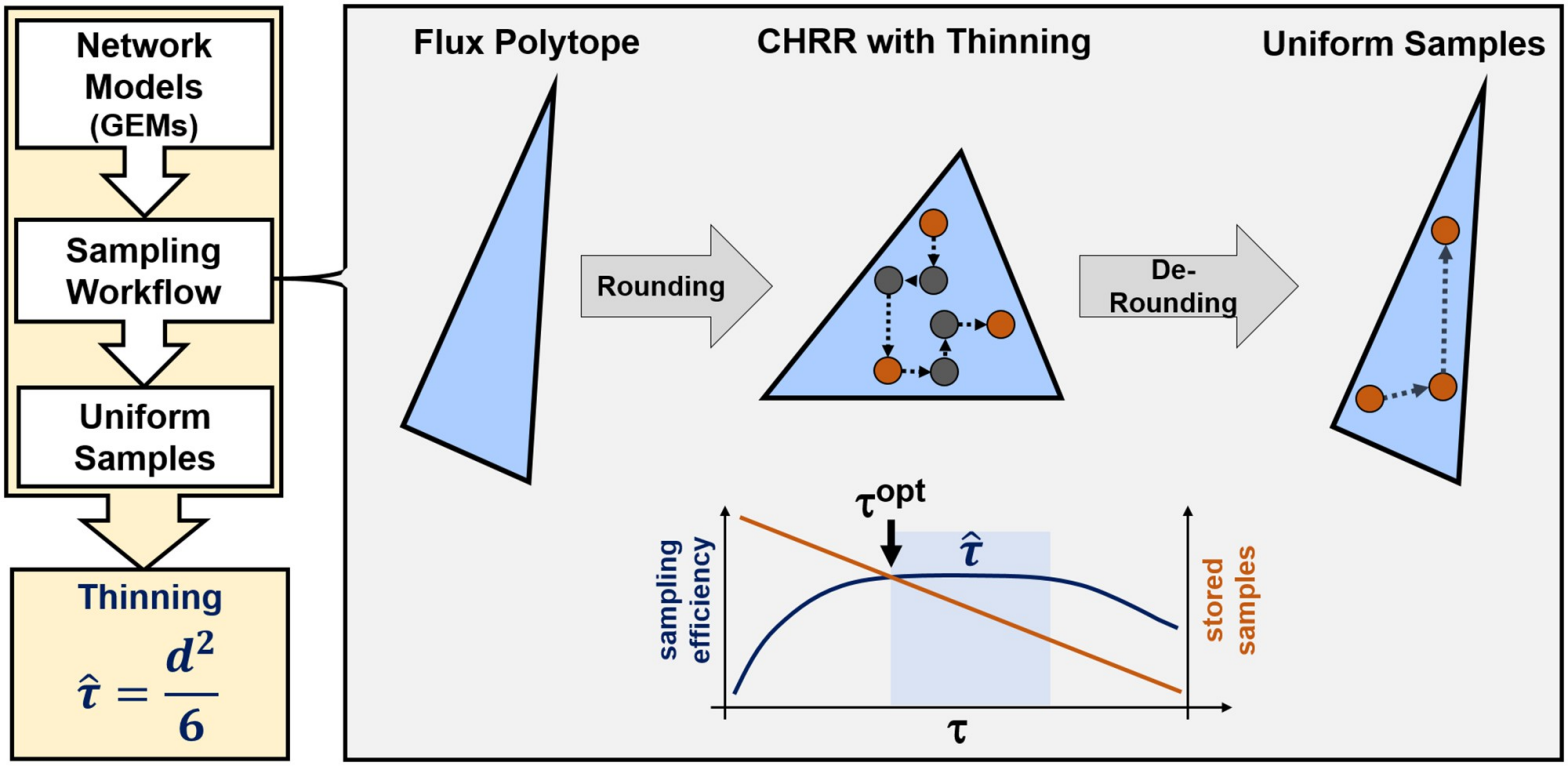
Overview of the CHRR tuning workflow. CHRR takes in a flux polytope that is sampled after bringing the polytope in a more favorable shape (rounding). By thinning, samples (grey) are deliberately dropped before transforming the remaining samples back to the original space (de-rounding). Finally, the samples (red) represent the uniform samples of the given GEM. Thinning is performance critical for CHRR, because de-rounding implies costs per-sample. Between the extremes of discarding too many samples and spending too much compute resources for de-rounding highly-correlated samples, an optimal thinning constant *τ*^*opt*^ exists. By running benchmarks for different GEMs and thinning constants, we derive a guideline for choosing $\tilde{\tau }$, close enough to *τ*^*opt*^ in efficiency, to sample unseen GEMs quickly.

Recognizing the “need for speed” to sample large-scale GEMs, in this work, we study the impact of thinning for CHRR systematically from the perspective of practical sampling efficiency, based on principled statistical criteria (cf. Fig 1). By analyzing simplices and GEMs with widely varying dimensionality, we demonstrate that CHRR is one of the rare cases for which thinning boosts sampling efficiencies, measured in terms of *ESS*/*t*, and prove current thinning practices computationally wasteful. From our benchmarks, we derive simple, yet effective and statistically rigorous guidelines to effectively and efficiently tune CHRR, which we verify using large-scale GEMs such as *Recon3D*. Our findings thus guide practical sampling efforts of COBRA models towards achieving more effective samples with less compute resources, thereby updating previous wisdom [42].

## 2 Methods and models

We start with a brief background on UCPS, describe the pre- and post-processing of flux polytopes relevant for the CHRR sampling procedure, list test and validation problems, and give details about MCMC diagnostic checks along with implementation details.

### 2.1 Convex GEM flux polytopes and their pre- and post-processing

GEMs are stoichiometric network models that are compiled from genomic information and biochemical knowledge [50]. By mass balancing at steady-state, linear inequality systems are derived from these models for the *D* unknown metabolic reaction rates (fluxes) $\nu \in R^{D}$:

$$\begin{array}{c} A_{\text{eq}}^{\prime }\cdot \nu =b_{\text{eq}}^{\prime } \end{array}$$ (2)

where $A_{\text{eq}}^{\prime }\in R^{n_{eq}^{\prime }\times D}$ is the (extended) stoichiometric matrix, and $b_{\text{eq}}^{\prime }\in R^{n_{eq}^{\prime }}$ contains the time derivatives of the (intra- and extracellular) metabolite concentrations. As a convention, we use the symbol “⋅” for the matrix-vector product. In addition, the fluxes *ν* are subject to linear inequalities originating from physiologically motivated lower and upper limits on their values:

$$\begin{array}{c} A_{\text{in}}^{\prime }\cdot \nu <b_{\text{in}}^{\prime } \end{array}$$ (3)

with $A_{\text{in}}^{\prime }\in R^{n_{in}^{\prime }\times D}$ the constraint matrix and $b_{\text{in}}^{\prime }\in R^{n_{in}^{\prime }}$ contains the flux bounds. The convex polytope (Eqs 2 and 3) is constructed such that it is always bounded in all flux directions, otherwise the uniform sampling problem is ill-defined. Since $A_{\text{in}}^{\prime }$ typically contains redundant constraints that cause numerical instabilities in the sampling workflow, in a first pre-processing step, the redundant inequalities are eliminated from Eq (3), giving

$$\begin{array}{c} A_{\text{in}}\cdot \nu <b_{\text{in}} \end{array}$$ (4)

with $A_{\text{in}}\in R^{n_{in}\times D}$ the constraint matrix and $b_{\text{in}}\in R^{n_{in}}$ Then, inequalities that are effectively equalities (fluxes bound to ranges equal or smaller than 1e-7) are identified and reformulated as equalities, which creates the modified stoichiometric system

$$\begin{array}{c} A_{\text{eq}}\cdot \nu =b_{\text{eq}} \end{array}$$ (5)

with $A_{\text{eq}}\in R^{n_{eq}\times D}$ and $b_{\text{eq}}\in R^{n_{eq}}$. Together, Eqs 4 and 5 define the *D*-dimensional effective convex-bounded flux polytope of the given GEM:

$$\begin{array}{c} P_{D}=\{\nu \in R^{D}|\;A_{\text{eq}}\cdot \nu =b_{\text{eq}}\wedge A_{\text{in}}\cdot \nu <b_{\text{in}}\} \end{array}$$ (6)

We note that by the pre-processing $P_{D}$ is slightly smaller than its original counterpart defined by Eqs (2) and (3). However, because the threshold, below which inequalities are interpreted as equalities, is selected to be orders of magnitude smaller than the achievable flux precision, the impact of the shrinkage on the flux values is in fact marginal. Instead, the beneficial effect on the numerical stability of the sampling algorithm outweighs the risk of losing relevant flux information [44].

Next, the equalities (5) are exploited to reduce the dimension of the sampling problem, without changing the polytope volume, by expressing the polytope $P_{D}$ in terms of independent, still interpretable flux coordinates $\nu^{\text{indep}}\in R^{d},\;d\leq D$ [51]. Therefore, we determine a basis $K\in R^{D\times d}$ of the null space of the modified stoichiometric system (5), subject to the inequality constraints (4):

$$\begin{array}{c} A_{\text{in}}\cdot \left(K\cdot \nu^{\text{indep}}+\nu_{0}\right)<b_{\text{in}} \end{array}$$ (7)

where *ν*_0_ is a particular solution within the flux polytope $P_{D}$. A typical choice for *ν*_0_ is the Chebyshev center of $P_{D}$. By going from Eqs (4) and (5) to Eq (7), all equalities are eliminated from $P_{D}$ in Eq (6), resulting in the dimension-reduced flux polytope $P_{d}$:

$$\begin{array}{c} P_{d}=\{\nu^{\text{indep}}\in R^{d}|\;A\cdot \nu^{\text{indep}}<b\} \end{array}$$ (8)

where $A=A_{\text{in}}\cdot K\in R^{n_{in}\times d}$ and $b=b_{\text{in}}-A_{\text{in}}\cdot \nu_{0}\in R^{n_{in}}$ with *ν*_0_ and *n*_*in*_ > *d* in all but toy networks. Hitherto, we call *d* the *effective* model dimension. As an example, *Recon3D*, as downloaded from BiGG, has 10, 600 bounded reactions. After dimension-reduction and flux coordinate transformation, *n*_*in*_ = 11, 195 inequality constraints and *d* = 4, 861 effective dimensions remain.

Flux polytopes are known to have anisotropic shapes, a characteristic that slows down the mixing of MCMC sampling schemes (see [22] for details). To curb for this, dimension-preserving so-called rounding transformations have been proposed that transform the polytope $P_{d}$ into a flux polytope $P_{d}^{\text{round}}$ having a more isotropic shape [22]. For more technical discussions of the isotropy of convex polytopes, we refer to [39, 52, 53]. In this work, using the approach in [44], we determine the linear rounding transformation $R\in R^{d\times d}$ by computing the Maximum Volume Ellipsoid (MVE) inscribed in $P_{d}$ [54]. Specifically, the inverse mapping *R*^−1^ is determined such that it maps the MVE to the unit sphere. The rounded polytope $P_{d}^{\text{round}}$ is then given by:

$$\begin{array}{c} P_{d}^{\text{round}}=\{\nu^{\text{round}}\in R^{d}|\;A_{\text{R}}\cdot \nu^{\text{round}}<b\} \end{array}$$ (9)

with $A_{\text{R}}=A\cdot R^{-1}\in R^{n_{in}\times d}$. Finally, any *d*-dimensional independent flux vector *ν*^round^ in the rounded space has to be mapped back to a flux vector *ν* in the original *D*-dimensional flux space to be biologically interpretable, using the affine back-transformation *T* : *ν*^round^ ↦ *ν* given by:

$$\begin{array}{c} \nu =K\cdot R^{-1}\cdot \nu^{\text{round}}+\nu_{0} \end{array}$$ (10)

### 2.2 Coordinate Hit-and Run with Thinning

Thinning is an integral part of many MCMC algorithms. We hitherto denote our implementation of CHRR [42] that makes deliberate use of thinning by CHRRT. Algorithm 1 gives a pseudocode description of the CHRRT algorithm. With a UCPS task at hand, defined by Eqs (4) and (5), first the rounded polytope $P_{d}^{\text{round}}$ is determined (L 1). Starting from an initial flux state, samples in $P_{d}^{\text{round}}$ are iteratively generated by constructing chords along the coordinates (L 6–10). Fixed frequency thinning is applied with thinning constant *τ* (L 11). Lastly, every sample that is not discarded due to thinning is transformed back to the full dimensional flux space $P_{D}$ (L 12).

**Algorithm 1** Coordinate Hit-and-Run with Rounding and Thinning (CHRRT)

**Input**:

 Equality constraints: *A*_eq_ ⋅ *ν* = *b*_eq_

 Inequality constraints: *A*_in_ ⋅ *ν* ≤ *b*_in_

 Number of samples to store: *N*

 Thinning constant: *τ*

 Feasible starting state: $\nu_{0}\in P_{D}$

**Result**:

 *N* uniformly distributed flux samples $\nu_{1},...,\nu_{N}\in P_{D}$

**Procedure**:

1: determine dimension-reduced polytope $P_{d}$

2: compute rounding transformation *R* and determine rounded polytope $P_{d}^{\text{round}}$

3: transform starting state: $\nu_{0}^{\text{round}}\leftarrow T^{-1}\left(\nu_{0}\right)$

4: *i* ← 0                ⊳ Label iteration of CHRRT with *i*

5: **while** number of stored samples < *N* **do**

6:  *k* ← uniform random coordinate ∈ {1, …, *d*}

7:  ⊳ compute distances from $\nu_{i}^{\text{round}}$ to borders of $P_{d}^{\text{round}}$:

8:  *d*_+_, *d*_−_ ← distances from $\nu_{i}^{\text{round}}$ to $\partial P_{d}^{\text{round}}$ along *k*^*th*^ coordinate

9:  $\lambda ∼U(d_{-},d_{+})$          ⊳ Sample step size *λ* uniformly from interval

10:  $\nu_{i+1}^{\text{round}}\leftarrow \nu_{i}^{\text{round}}+\lambda \cdot e_{k}$      ⊳ Update in direction of unit vector *e*_*k*_

11:  **if** *i* modulo *τ* is 0 **then**

12:   store result of $T(\nu_{i+1}^{\text{round}})$   ⊳ Back-transform sample to original flux space $P_{D}$

13:  **end if**

14:  *i* ← *i* + 1

15: **end while**

Concerning the computational costs of CHRRT, determining an internal point *ν*_0_ and computing the rounded polytope $P_{d}^{\text{round}}$ are one-time costs, both being independent of the numbers of samples. We therefore do not further consider these costs in this work. The most expensive operation per sample is the back-transformation *T* that maps the generated sample to the original polytope (Algorithm 1, L 12). The back-transformation amounts to a dense matrix-vector multiplication, with worst case costs of $O(n_{in}\cdot d)$ per sample. All other operations are element-wise additions, multiplications, or divisions with per-sample costs of O(d).

### 2.3 Test problems

We consider two types of test problems, simplices representing well-defined and easy to scale problems, and GEMs being our main concern. A *d*-dimensional simplex $S_{d}$ is defined by:

$$\begin{array}{c} S_{d}=\left\{x\in R^{d}\;|\;\sum_{i=1}^{d}x_{i}\leq 1,\;x_{i}\geq 0\;\forall i\leq d\right\} \end{array}$$ (11)

Being easily scalable to different dimensions, simplices are ideal to study the dependency of sampling complexity and polytope dimension. Different to GEMs, simplices are constructed by non-redundant constraints and, therefore, do not need to be dimension-reduced. However, as for GEMs, polytope rounding improves the geometric isotropy of simplices. Thus, Eq (10) remains valid, with the matrix *K* set to identity. In this work, simplices in 64, 256, 1,024, and 2,048 dimensions were benchmarked.

Besides simplices, we selected eleven GEMs of varying sizes for this study, eight for training our thinning guideline, and three (*Yeast8*, *ecYeast8*, *Recon3D*) for validation purposes. Effective polytope dimensions range from 24 to 4,861. In contrast to simplices, for GEMs there is no consistent relationship between the number of reactions, the number of constraints, and the effective polytope dimension *d*. We refer to Table A in S1 Appendix for more details on the test problems.

### 2.4 MCMC convergence diagnostics

Assessing whether a (sub)sequence of MCMC samples has converged to the target distribution is not only vitally important in practical inference, but also critical for reliable benchmarking sampling performances. The *ESS* and rank-normalized $\hat{R}$ values are computed for each of the *D* fluxes separately and set to the minimum/maximum value of these, i.e., considering the worst-case. We compute the *ESS* in the original flux space, because this is the space, for which we are interested in statistics about estimates, such as the mean flux. Particular care must be taken here, since the estimated *ESS* is typically noisy and may therefore be unreliable for sample sets that are far from being converged. We here follow the advice given by [55] to use four independent chains and compute the rank-normalized $\hat{R}$ diagnostic along with the *ESS* to test for convergence. Vehtari et al. [55] argue that the estimation of $\hat{R}$ and *ESS* requires reliable estimates of the variances and autocorrelations of the samples, that is, the estimation of $\hat{R}$ and *ESS* is only reliable, if the *ESS* of a set of samples is sufficiently large. Therefore, in our study, the threshold for convergence is set to $\hat{R}<1.01$ and *ESS* > 400, which is suitable to a setup with four independent Markov chains. The limit of 400 for the *ESS* is derived by considering that for a single chain, each of the parts of the split chain (50/50 split) is required to contain *ESS* > 50, which amounts to a total of at least 400 when considering multiple, independent chains [55]. Exemplary *ESS* and rank normalized $\hat{R}$ values are shown in Figs B and C in S1 Appendix.

### 2.5 Implementation details

GEMs were downloaded (Table A in S1 Appendix for details) in SBML (Systems Biology Markup Language) format [56], and plugged into PolyRound v0.1.8, the state-of-the-art python package for polytope rounding [44]. The highly optimized polytope sampling library HOPS v3.0.0 was used for MCMC sampling [46]. CHRRT, as implemented in HOPS, was used for all benchmark runs to ensure their comparability. In each sampling run, four parallel chains were used according to recommendations by Vehtari et al. [55]. Samples, the rounded polytopes, and the accompanying transformations were stored. For each flux, trace plots were visually examined, the maximum rank-normalized $\hat{R}$ metric, and the minimal *ESS* over all fluxes were computed to check that MCMC convergence criteria are met as described in Sec. 2.4. Calculations are performed using arviz v0.12.1 [57]. All numerical experiments were run on an AMD Ryzen 9 5950X 16-Core Processor.

## 3 Results and discussion

After motivating the idea of using thinning in CHRR to reallocate computational resources, we quantify achievable sampling efficiencies for the test problems. From that, we derive simple and useful guidelines for optimized thinning choice, which we compare to previous advice using three validation problems.

### 3.1 The role of thinning for CHRR

In constraint-based metabolic modeling, some studies using CHRR report thinning constants of 100, 1000, and 10,000 [4, 43]. However, these works neither do provide guidance for a suitable selection of *τ* for a sampling problem at hand, nor do they discuss the implications of the different choices of *τ*. On the other hand, Haraldsdóttir et al [42] pointed out that *τ* should be selected depending on the problem dimension. Specifically, the authors suggest setting *τ*, as a rule of thumb, to eight times the squared polytope dimension, stating that this choice ensures statistical independence of the thinned samples. However, in this work no derivation or their rule or further underpinning of their argument is given.

Spurred by the seminal theoretic work of Geyer [48], we set out to investigate whether the sampling efficiency of CHRR benefits from thinning. The question is whether, for a fixed computational budget, CHRR with *τ* > 1 convergences faster than unthinned CHRR with *τ* = 1. To this end, we consider the *ESS* of a set of *N* samples in relation to the time cost of producing the samples, *t*_*N*,*τ*_. The time cost *t*_*N*,*τ*_ is given by the sum of the time costs *t*_update_ for producing *N* ⋅ *τ* states $\nu_{i}^{\text{round}}$ in Algorithm 1 (L 6–10), and the time cost *t*_transform_ of transforming the *N* samples back to the original flux space (Algorithm 1, L 12):

$$\begin{array}{c} t_{N,\tau }=N\cdot (\tau \cdot t_{\text{update}}+t_{\text{transform}}) \end{array}$$ (12)

Geyer argued that for thinning to be beneficial, the time cost *t*_*N*,*τ*_ has to grow slower with *τ* than the *ESS*, indicating that thinning is not advantageous for every combination of sampling problem and MCMC algorithm [48]. In this vein, Link et al. criticize the routine application of thinning for applications in ecology, where thinning is often detrimental to sampling efficiency [49].

**Fig 2:**
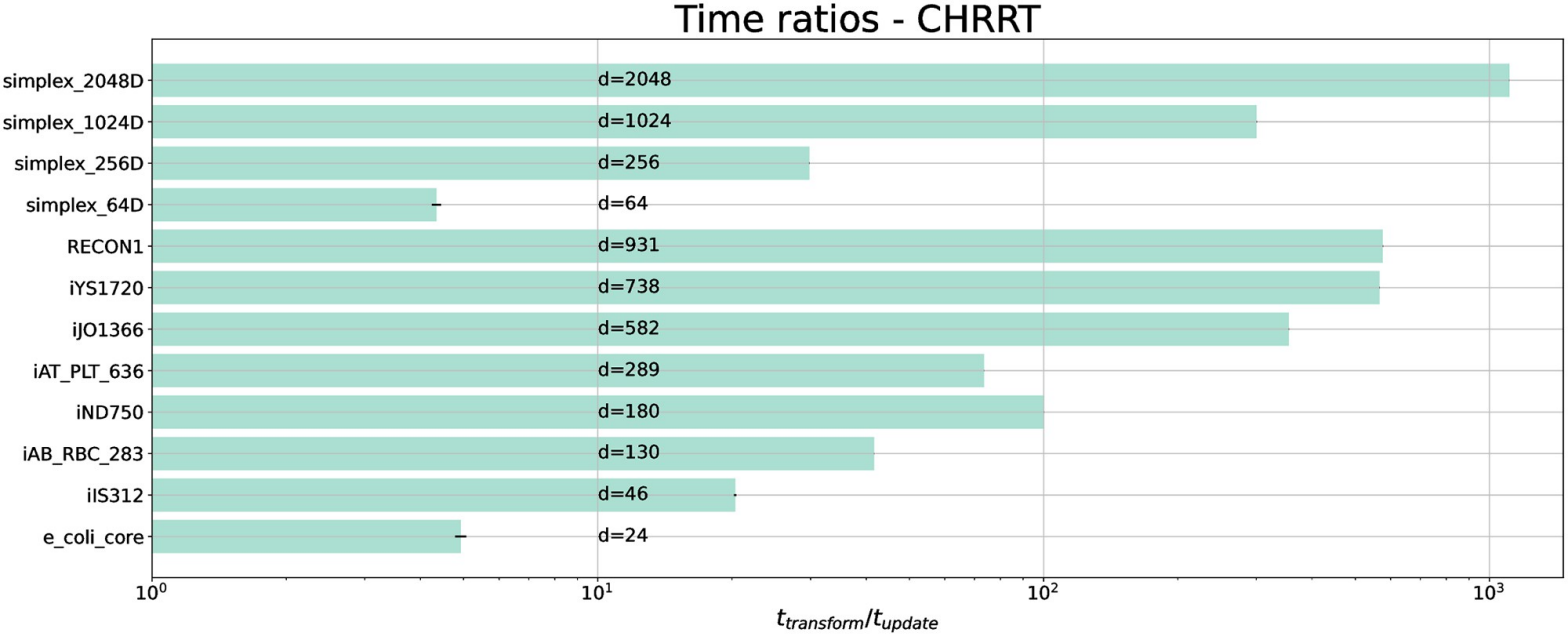
Mean time ratios of one back-transform *t*_transform_ vs. one CHRRT update *t*_update_ for simplices and GEMs (horizontal axis scaled logarithmically). Ratios indicate that in all cases, the cost to transform a sample back to the original flux space exceeds the cost of generating a sample by far. In all but the smallest test problems, the error bars of four runs are too small (< 1%) to be visible. For the simplices the ratio increases quadratically with dimension (0.00024 ⋅ *d*^2^ + 0.044 ⋅ *d* + 1.4). For GEMs, there is a strong trend towards an increase in ratio with model dimension. Details on the models are found in Table A in S1 Appendix.

To investigate the role of thinning for UCPS using our CHRRT implementation, we measured *t*_update_ and *t*_transform_ for a set of synthetic and real test problems (Table A in S1 Appendix). For both time costs, we took the wall-clock times and averaged them over the number of times each operation was applied. The resulting time ratios *t*_transform_/*t*_update_ are shown in Fig 2. Among all test problems, the time ratios for only the two models with the fewest dimensions, i.e., the 64-dimensional simplex and the 24 dimensional *E. coli* core model, are below ten. For the remaining investigated models, *t*_transform_ is up to three orders of magnitude larger than *t*_update_. Precisely, for the simplices, the time ratio increases quadratically with dimension. We also observed a stark increase in the ratio for GEMs. Different to simplices, however, with GEMs additional factors contribute to the time ratios, such as the number of fluxes *D* (more fluxes increase *t*_transform_) and the number of constraints *n*_*in*_ (more constraints increase *t*_update_), resulting in a less succinct relation between effective model dimensions and time ratios.

Across all test problems, *t*_transform_ is generally considerably larger than *t*_update_, with a time ratio of at least four for the smallest tested models, but which starkly increases with the dimension of the sampling problem. Given such large time ratios, it is plausible to expect thinning to be beneficial for the sampling efficiency of CHRR in the context of UCPS. However, only by considering the relation between the time ratios and the autocorrelation mediated by the *ESS*, which is specific to the combination of model characteristic and CHRR, we are able to find out if thinning actually is practically beneficial. In conclusion, low autocorrelation should not be the only deciding factor when suggesting rules of thumb for thinning constants.

### 3.2 Benchmarking CHRR from the perspective of thinning

To study how the performance of CHRR depends on thinning for the two classes of test problems, we benchmarked the sampling efficiency, in terms of *ESS/t*, for a wide variety of thinning constants. To find thinning constants that yield high *ESS*/*t* values, pre-runs with *τ* = *d* were performed for each test problem. From the pre-runs, we estimated appropriate ranges of problem-specific thinning constants and selected a set of at least five *τ*’s distributed over that range.

**Fig 3:**
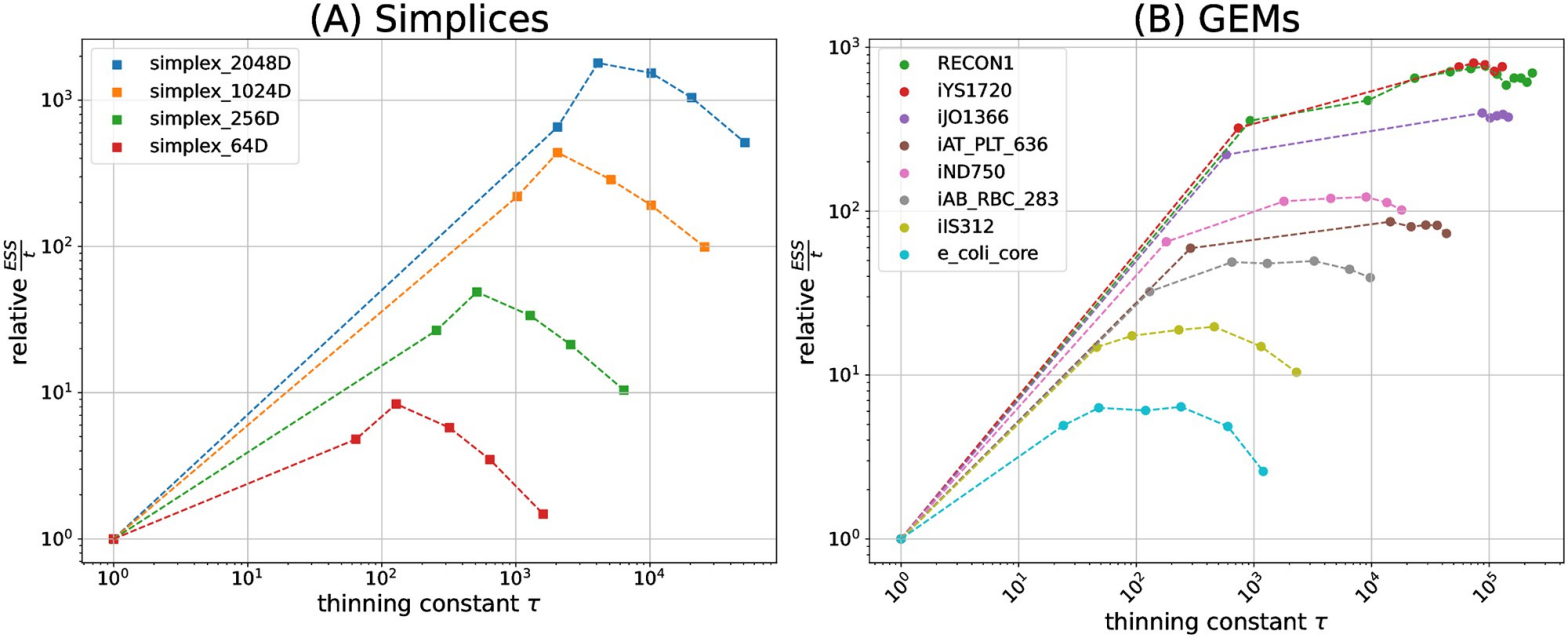
Double logarithmic plot of sampling efficiencies, relative to the unthinned sampling efficiencies, for selected thinning constants *τ*. The corresponding absolute *ESS*/*t* values are provided in Fig A in S1 Appendix. (**A**) Benchmark results for simplices. The peak efficiency is reached for a thinning constant $\hat{\tau }=2\cdot d$, with *d* being the number of effective dimensions. Over all, the maximum speed-up is achieved for the 2,064-dimensional simplex, being 1,798 times faster in terms of *ESS*/*t* compared to unthinned CHRR. (**B**) Benchmark results for GEMs. Setting *τ* > 1 generally improves the *ESS*/*t*. For increasing *d*, larger thinning constants further boost the sampling efficiency. In all cases, the emerging plateaus indicate that the optimal *ESS*/*t* is not overly sensitive to the precise choice of the thinning constant.

For simplices and GEMs, Fig 3 shows the obtained *ESS*/*t*, relative to the unthinned CHRR baseline. The plots fan out in a series of curves for the test problems with different model dimensions. As a rule, for higher dimensions, higher relative *ESS*/*t* values are achieved. All curves share the characteristic that with increasing *τ*, the relative *ESS*/*t* first increases before dropping again. The peak indicates the optimal thinning constant choice, specific to the investigated test problem. While for simplices pronounced peaks emerge, the curves for GEMs show plateaus, indicating that a range of thinning constants perform equally well. This apparent insensitivity in *ESS*/*t* performance for GEMs is convenient, since it implies that hyperparameter tuning of *τ* does not need to be precise for achieving near optimal *ESS*/*t* values, if its value is set to a suitable order of magnitude. Clearly, thinning constants at the lower end of the plateau are to be preferred due to a better resource usage (see below).

**Fig 4:**
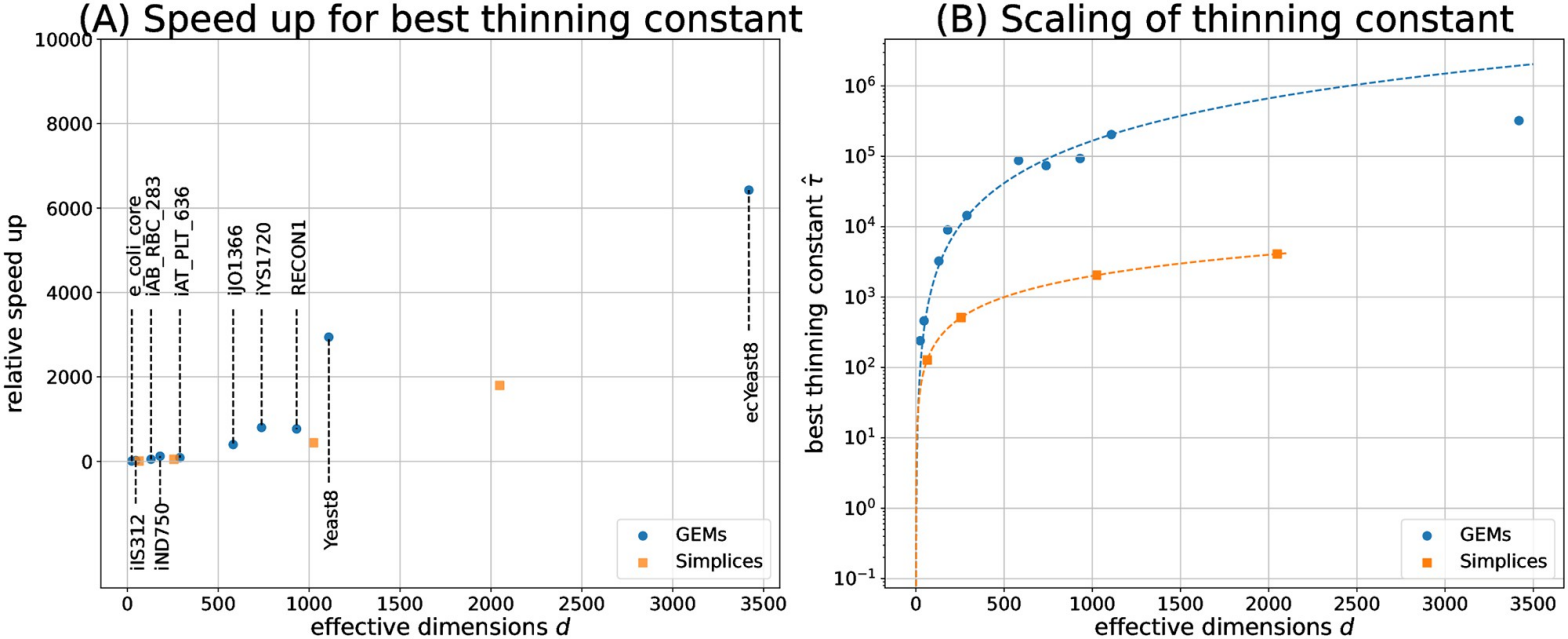
Performances of best thinning constants. (**A**) Speed-ups achieved for the best thinning constants $\hat{\tau }$, compared to the unthinned baseline, for simplices (orange squares) and GEMs (blue cicles). (**B**) Scaling of the best thinning constant given the effective model dimensions *d* (vertical axis scaled logarithmically). Guidance for the selection of useful thinning constants $\hat{\tau }$ for GEMs (blue line) and simplices (orange line) is obtained by regression. For problem dimensions above 500, $\hat{\tau }$ is about two orders of magnitude larger for GEMs than for simplices.

To study how the optimal (in terms of sampling efficiency) thinning constant relates to the problem dimension, we then quantified the speed-up of CHRRT for the apparently best thinning constant, hitherto denoted $\hat{\tau }$, among the set of tested constants. Results are shown in Fig 4A. For simplices, relative speed-ups grow roughly to the square of the polytope dimension. The relative speed-ups achieved for GEMs also grow with effective model dimension, with values ranging from 6 for the *E. coli* core model up to 770 for *Recon1*. For the latter, CHHRT with $\hat{\tau }$ required only 0.32 s to converge and reach an *ESS* of 4,954, the unthinned variant required almost 80 times longer (25 s) to converge, while it reached an *ESS* of only 507. Notably, the speed-ups observed for GEMs surpass those observed for simplices of similar dimensions. Hence, our benchmarks show that the beneficial effect of thinning, when appropriately tuned, is not only plausible in theory, but it is also of high practical relevance for solving high-dimensional UCPS problems.

Since thinning drastically affects performances, our findings imply that the consequences of selecting *τ* values need to be taken into consideration when benchmarking novel aspiring UCPS algorithms against CHRR, as for example the truncated log-concave sampling with reflective Hamiltonian Monte Carlo [53] or the Riemannian Hamiltonian Monte Carlo in a Constrained Space [58].

### 3.3 Simple thinning guidelines for simplices and GEMs

We use the previously benchmarked GEMs and simplices as training data for deriving practical guidelines on how to configure thinning for CHRR for optimized performance. For *d*-dimensional simplices, the linear relation

$$\begin{array}{c} {\hat{\tau }}_{\text{simplex}}=2\cdot d \end{array}$$ (13)

explains the measured data perfectly. For GEMs, on the other hand, a quadratic relation of the form 0.164 ⋅ *d*^2^ matches the data well. Considering Fig 3B, indicating that optimal sampling performance is not overly sensitive to thinning constant choice, as a memorable rule of thumb for efficient GEM sampling, we propose:

$$\begin{array}{c} {\hat{\tau }}_{\text{GEM}}=\frac{d^{2}}{6} \end{array}$$ (14)

which only marginally deviates (less than 1.5%) from the fitted line.

Compared to the advice previously given by Haraldsdóttir et al. [42], i.e., 8 ⋅ *d*^2^, our GEM guideline advocates thinning constants that are a factor of 48 smaller. To put the difference between the former and our updated guideline into perspective, a 48-fold increase in *τ* means a 48-fold increase in CHRR update steps, of which the vast majority is discarded. As we have argued before, discarding samples can lead to an improvement of sampling efficiency, but only if the loss of information due to dropping samples is over-compensated by information from additional CHRR update steps. In addition, in Fig 3 we show that selecting the thinning constant too large risks missing the performance peak, which then wastes computational resources. In particular, we observe that a 48-fold increase in *τ* would lead to lower sampling efficiencies for eight out of the twelve test problems, because the performance peak would be clearly missed. For the remaining four test-problems, i.e., GEMs of higher dimensionality, we did not attempt to characterize the plateau around the best thinning constants, since this would be of little practical relevance. It is generally preferable to set the thinning constant to the minimal value on the plateau, because then less work is discarded, and more samples are stored than for larger thinning constants, while achieving the same *ESS*/*t*. Nonetheless, it is imaginable that the performance plateaus for the four largest GEMs stretch across an even wider range of thinning constants, meaning that the difference in *ESS*/*t* resulting from the previous rule of Haraldsdóttir et al. [42] and our guideline may turn out to be smaller.

While more work could be invested into fine-tuning $\hat{\tau }$ for the problem at hand, this risks investing more computational work than is eventually gained, because the estimation of *ESS* is noisy for short CHRR runs. In summary, we showed that a previous guideline for GEM sampling is suboptimal from the perspective of sampling efficiency. Instead, we promote a new GEM guideline, given by Eq (14), which empirically maximizes sampling performance without unnecessarily discarding computational work (green computing).

### 3.4 Result verification

To verify our proposed guideline for GEM sampling using CHRR, we applied it to three large, out-of-sample GEMs: *Yeast8* (1,108 dimensions), *ecYeast8* (3,4109 dimensions), as well as *Recon3D* (4,861 dimensions), the largest GEM currently available in the BiGG database [13]. All validation models were larger than the largest training model (*Recon1*), cf. Table A in S1 Appendix).

**Fig 5:**
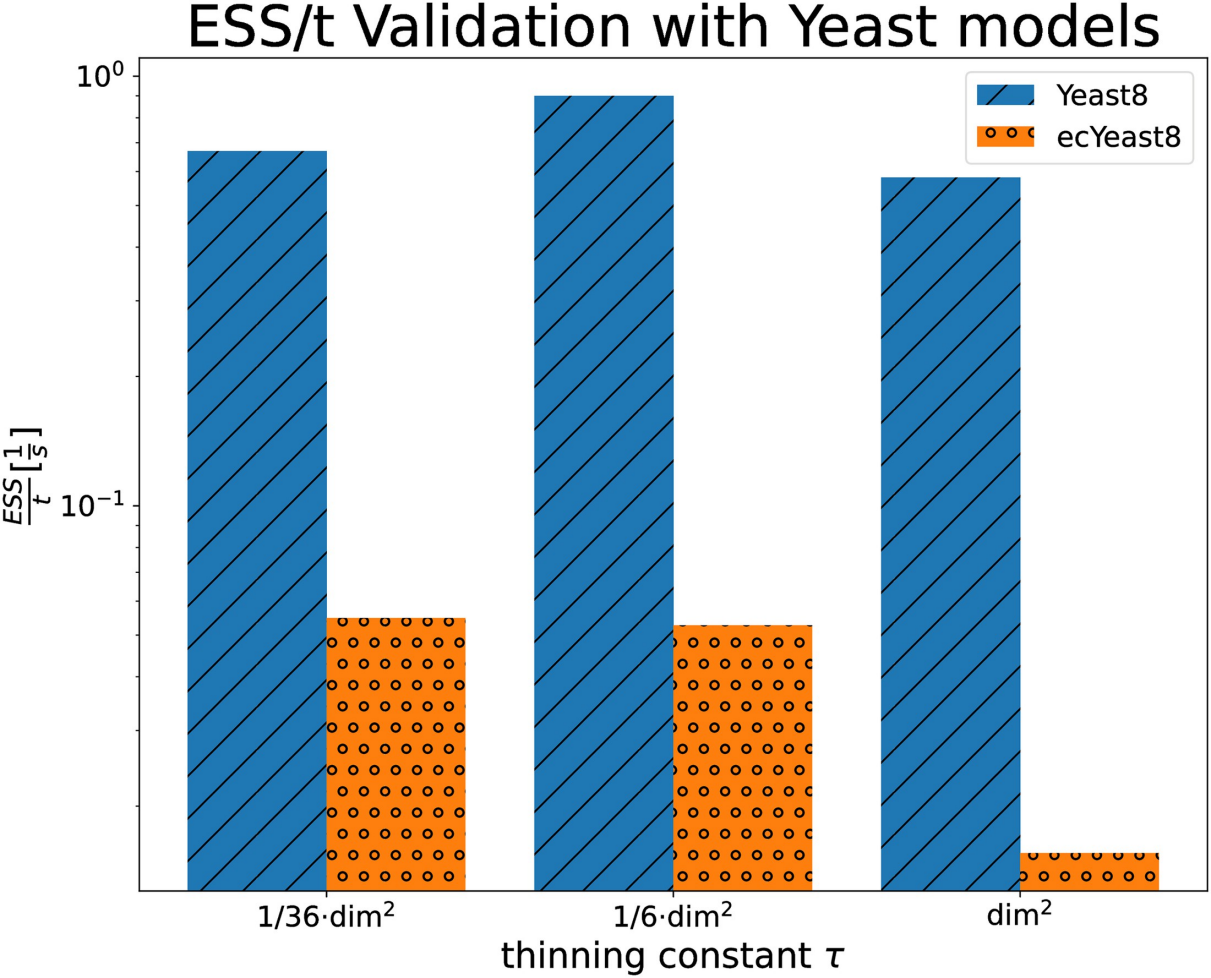
Measured *ESS*/*t* for a range of thinning constants *τ* for the 1,108 dimensional *Yeast8* (blue) and the 3,419 dimensional *ecYeast8* models. For *Yeast8*, the guideline $\hat{\tau }=d^{2}/6$ produced the highest performance, while for *ecYeast8* $\hat{\tau }=d^{2}/6$ was measured to be around 4% more efficient *ESS*/*t* = 0.0547 s^-1^ vs. *ESS*/*t* = 0.0527 s^-1^.

Given the large validation models, it is computationally infeasible to benchmark inefficient thinning constants (especially unthinned CHRR). Therefore, we need alternative ways to test, whether our guideline leads to optimized sampling. For the yeast models, *Yeast8* and *ecYeast8*, we benchmarked our guideline $\tau =\frac{d^{2}}{6}$, a smaller thinning constant $\tau =\frac{d^{2}}{36}$, and a larger thinning constant *d*^2^. This experimental setup allowed us to test, whether our guideline actually finds the (potentially wide) peak *ESS*/*t*. After checking for convergence, we measured the ESS/*t*, see Fig 5.

For *Yeast8*, setting *τ* according to our guideline clearly produced the best performance. For *ecYeast8* a six times smaller thinning constant was slightly more efficient (around 4%). However, choosing a six times larger thinning constant than our guideline decreased performance substantially. Because the *ESS*/*t* as a function of *τ* is not multimodal, we can conclude, that the 48 times larger *τ*, suggested previously [42], performs worse. The estimated time ratios for *Yeast8* (957) and *ecYeast8* (2090) let us conclude that very small thinning constants, such as *τ* = 1, are inefficient, because too much time would be spent on the back-transform. From these two test-cases, it is clear, that thinning should not be too small nor too large and that our guideline leads to practical performance.

For testing our guideline with the largest validation model, *Recon3D*, we needed to minimize the computational work even further, to achieve results in a reasonable time. We selected two thinning constants to test, namely our guideline, see Eq (14), and previous advice (*τ* = 8 ⋅ *d*^2^) [42]. By benchmarking these two thinning constants, we test, whether our guideline leads to an improvement over existing advice.

For the previous advice (*τ* = 8 ⋅ *d*^2^ = 8 ⋅ 4, 861^2^ = 189, 034, 568), we observed that CHRRT requires 1.1 h to produce and store one sample per chain. Because it is computationally infeasible to run *τ* = 8 ⋅ 4, 861^2^ to convergence, i.e., to obtain a representative number of samples, we estimate an upper bound on the efficiency of this thinning constant. Assuming optimistically that each of these samples is uncorrelated, i.e., *ESS* = *#stored samples*, we project that it takes approximately 110 h until convergence, because both parts of the split chain are required to reach an *ESS* of 50 [55]. For four parallel and independent chains, as in our benchmarks above and as advised [55], this corresponds, in the best of all cases, to an *ESS*/*t* of at most $\frac{4}{1.1h}=0.001$ s^−1^. Using the newly proposed guideline in Eq (14), CHRRT with a thinning factor of ≈ 3, 938, 220, it takes 27 h to generate 1, 000 samples per chain. Using four parallel chains, this setup results in an *ESS* of 726 with an $\hat{R}$ of ≈ 1.01. Therewith, our guideline yielded an *ESS*/*t* = 0.0075 s^−1^, which is 7.5 times more efficient than the overly optimistic upper bound we found for the previously advised rule, when both methods use four chains. In summary, the new guideline is not only more efficient, but it also produces a converged set of samples for storage in a fraction of the time. By using three validation models, we have empirically demonstrated that our guideline leads to efficient sampling, also for larger GEMs.

## 4 Conclusion and outlook

CHRR is the leading algorithm for sampling convex polytopes uniformly, having a single adjustable parameter, the thinning constant. In this work, we showed that the choice of the thinning constant has dramatic consequences on sampling efficiencies, which are instrumental for solving high-dimensional UCPS problems. Here, selecting an appropriate thinning constant can make the difference between sampling success and failure, thus, being the key to sample challenging network models to convergence. Especially for contemporary GEM sampling, it is therefore crucial to have a guideline at hand for the selection of the thinning constant that achieves near-optimal performance.

With a range of UCPS problems at hand, we studied the effect of thinning on CHRR sampling performance quantitatively, after explaining the statistical underpinnings of the efficiency metric (*ESS*/*t*). From our quantitative benchmarks, we derived simple guidelines for optimal CHRR thinning constant choice for simplices and GEMs, which we validated using three out-of-sample, larger GEMs. Applying these guidelines is not only beneficial for obtaining uniform samples of polytopes faster, but also helps to migrate UCPS towards green computing, as both the CPU time and the storage cost of samples are reduced. Concerning the question of how the computational requirements of CHRR with optimal thinning scale with effective model dimension, our numerical results reveal a quadratic and linear correlation for simplices and GEMs, respectively. Using the methods we have presented in our study, our guideline can be updated as new types of models and data become available.

Benchmarking new UPCS algorithms and comparing their performances with those of leading algorithms, such as CHRR, is important to advance the field and a topic of active research [53, 58]. Recognizing the substantial impact of thinning on the performance of CHRR, we advise performing comparisons with tuned thinning, and to report the used thinning constant, which will help their reproduction.

Replacing the sequential per-sample by a one-shot or batched back-transform (Algorithm 1, L 12) could be more efficient, as long as memory issues are not limiting (storing unthinned samples of large models, such as *Recon3D*, consumes prohibitively much memory). To combat memory bottlenecks, Stein thinning [59], a technique to optimally compress the MCMC output, may be applied before sample back-transformation. However, while this combination of techniques is promising, the implementation is not straightforward. In contrast, fixed-frequency thinning, as discussed in this work, is already implemented in many existing CHRR packages [4, 42, 46] and immediately boosts performance without requiring additional work.

Despite thinning being a common MCMC practice, it is still controversially discussed. Statisticians have pointed out that thinning is often not necessary and that it typically wastes computational resources, unless the cost of using the samples is high [48, 49]. Even then, Geyer argues that a thinning constant of two or three times the problem dimension should be suited in nearly all cases. Consequently, the conventional advice given to the MCMC practitioner is to not thin MCMC outputs, unless memory or post-processing capabilities are practically limiting. By showing that CHRR stands out among MCMC algorithms in that thinning is critical to performance for sampling high-dimensional convex polytopes, such as GEMs, our study encourages researchers to systematically examine conventional MCMC advice in their specific application domain.

## Supporting information

S1 Appendix Supporting information. Overview of benchmark problems, measured sampling efficiencies, convergence diagnostics, and exemplary flux distributions for selected GEMs.
(PDF)
