# Supplementary material for: Practical sampling of constraint-based models: Optimized thinning boosts CHRR performance
Source: PLoS Comput Biol. 2023 Aug 11;19(8):e1011378. doi: 10.1371/journal.pcbi.1011378 (PMC10446239; doi:10.1371/journal.pcbi.1011378)
Supplement: S1 Appendix — Overview of benchmark problems, measured sampling efficiencies, convergence diagnostics, and exemplary flux distributions for selected GEMs. (PDF) [file pcbi.1011378.s001.pdf]

Supplementary Information for

Practical sampling of constraint-based models: Optimized thinning boosts CHRR performance

Johann F. Jadebeck [1,2], Wolfgang Wiechert [1,2] and Katharina Nöh [1,*]

[1] Institute of Bio- and Geosciences, IBG-1: Biotechnology, Forschungszentrum Jülich, 52425 Jülich, Germany

[2] Computational Systems Biotechnology (AVT.CSB), RWTH Aachen University, 52062 Aachen, Germany

[*]Correspondence: k.noeh@fz-juelich.de

1 Benchmark models

Table A: **Benchmark models** for tuning and validation of the thinning guideline. Overview of models and simplices and their usage. The number of constraints gives the number of non-redundant inequalities after preprocessing with PolyRound [1] as described in the main text (cf. Sec. Materials and models).

Model	Type	#reactions	# effective polytope dimensions d	#constraints n_{in}	Usage	Reference
e_coli_core	GEM	95	24	36	Training	[2]
iIS312	GEM	519	46	137	Training	[3]
iAB_RBC_283	GEM	469	130	188	Training	[4]
iND750	GEM	1,266	180	273	Training	[5]
iAT_PLT_636	GEM	1,008	289	593	Training	[6]
iJO1366	GEM	2,583	582	752	Training	[7]
iYS1720	GEM	3,357	738	962	Training	[8]
RECON1	GEM	3,741	931	2,120	Training	[9]
Yeast8 (v8.6.2)	GEM	4,063	1,108	1,760	Validation	[10]
ecYeast8 (v8.3.4)	GEM	8,143	3,419	6,295	Validation	[10]
Recon3D	GEM	10,600	4,861	11,195	Validation	[11]
simplex_64D	Simplex		64	65	Training	
simplex_256D	Simplex		256	257	Training	
simplex_1024D	Simplex		1,024	1,025	Training	
simplex_2048D	Simplex		2,048	2,049	Training	

2 Measured ESS/t

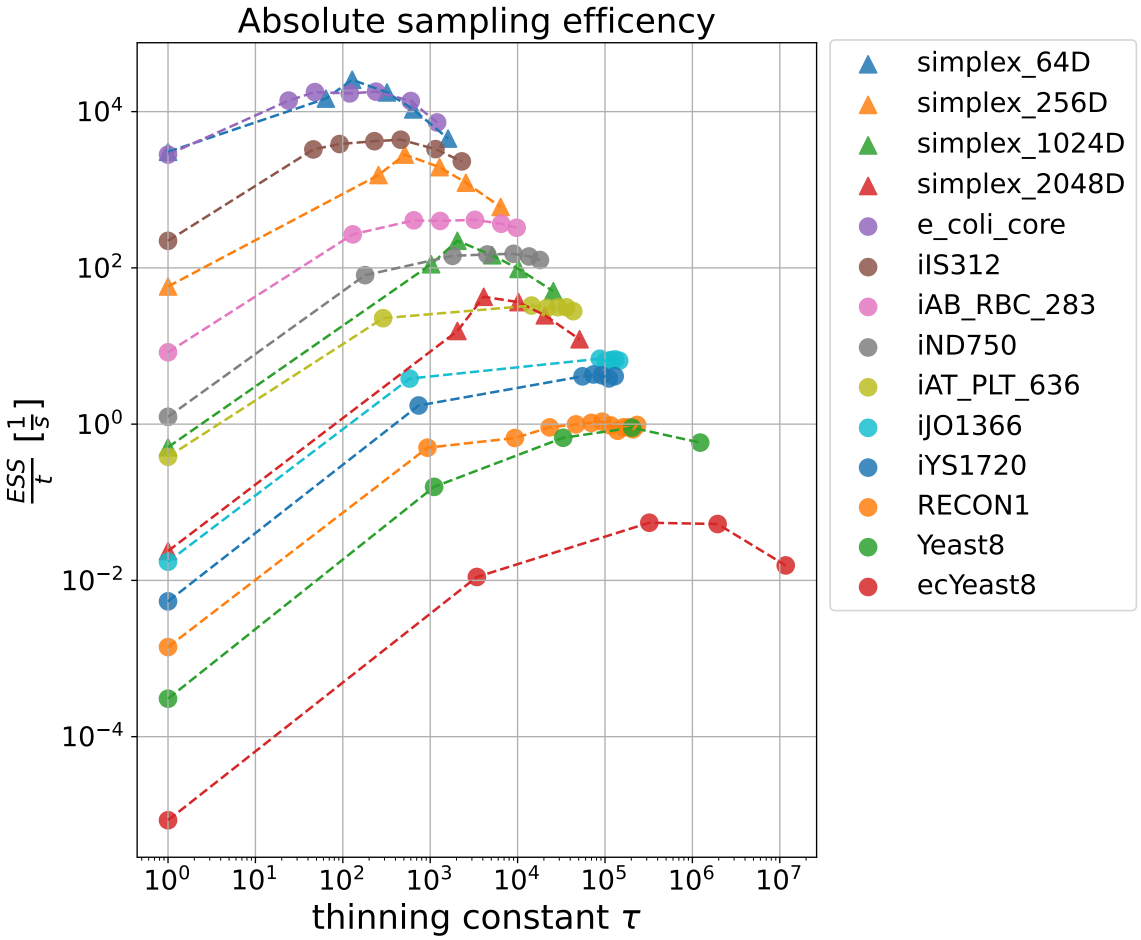

Figure A: **Measured** ESS/t**.** Double logarithmic plot of absolute sampling efficiencies as measured by ESS/t for selected thinning constants τ. ESS is determined by taking the minimum of the flux-specific values, i.e., $\min\{ESS_i\}_{i=1,...,D}$. Note that CHRRT for simplices achieves a higher absolute performance compared to sampling GEMs with similar dimensions. As an example, the best thinning constant $\hat{\tau}$ for *simplex_2048D* is comparable to the best thinning constant for the *iAT_PLT_636* model, which has 582 dimensions. Comparing the absolute ESS/t across various problems demonstrates that the effective dimensionality of the polytope is one, but not the only component determining sampling complexity.

3 Measured *ESS*

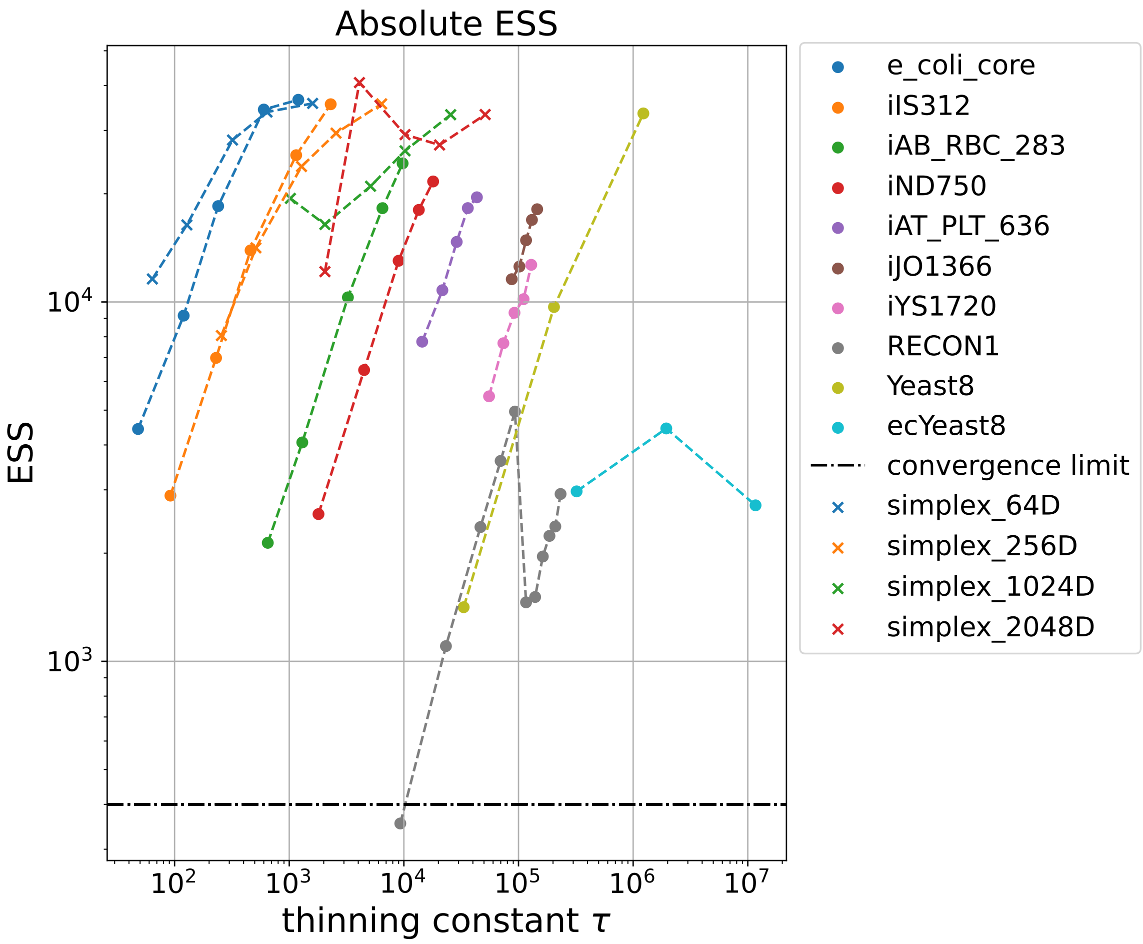

Figure B: **Measured *ESS*.** Double logarithmic plot of measured *ESS* for selected thinning constants τ. In almost all cases, the simulations were set up such that the minimum required $ESS = 400$ for all fluxes was achieved. Notable exceptions are a thinning constant of approximately 10^4 for *Recon1*, where the ESS was so close to 400 that it is unlikely to bias the results. Furthermore, the convergence was clearly achieved in the interesting regions of the thinning constant, see Fig A.

4 MCMC convergence diagnostics

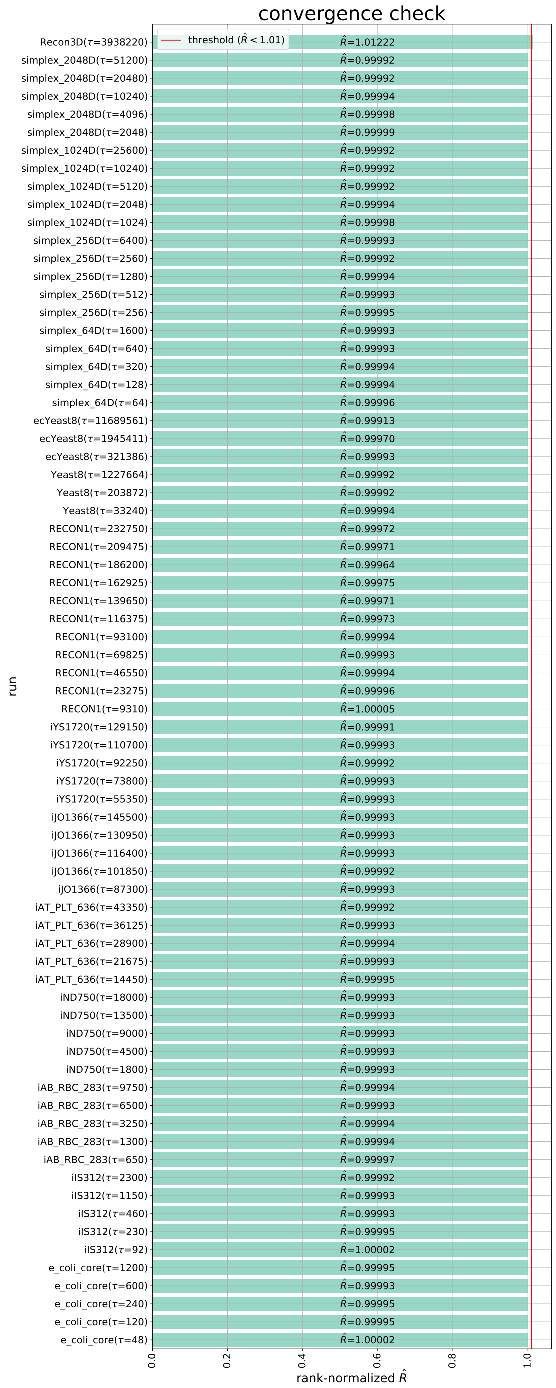

Figure C: **Measured rank-normalized** $\hat{R}$. A value close to one indicates convergence. In the case of *Recon3D*, the value is slightly above the threshold of 1.01 suggested in [12]. However, since the *ESS* is high (around 750), it is unlikely that this has a sizable impact on our results.

5 Flux-specific ESS

ESS values are given as the minimal value over all flux parameters, i.e., $\min\{ESS_i\}_{i=1..D}$. Here, we report the flux-specific ESS values for selected models. Note that the measured ESS does not directly map to sampling efficiency, because the different thinning constants τ were not run equally long. Flux-specific ESS plots for the remaining models in Table S.1 are available in our code repository (https://jugit.fz-juelich.de/IBG-1/ModSim/Fluxomics/chrrt).

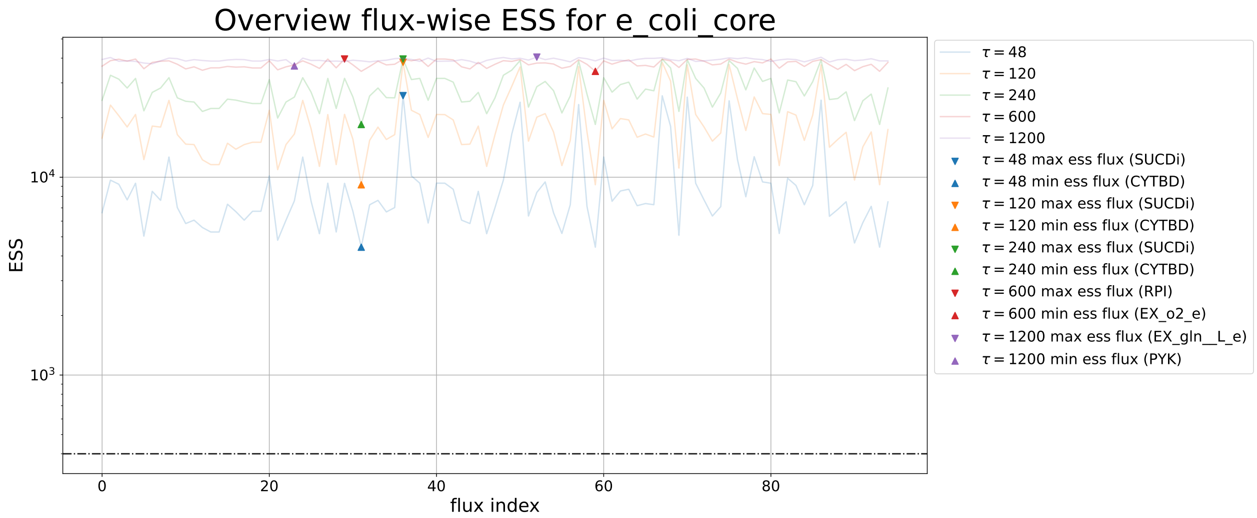

Figure D: **Flux-specific *ESS* a small core model of *E. coli* (*e_coli_core*) for different thinning constants τ.** With increasing thinning constant, the ESS equalizes across the fluxes in the sense that the difference between the highest and lowest ESS is reduced.

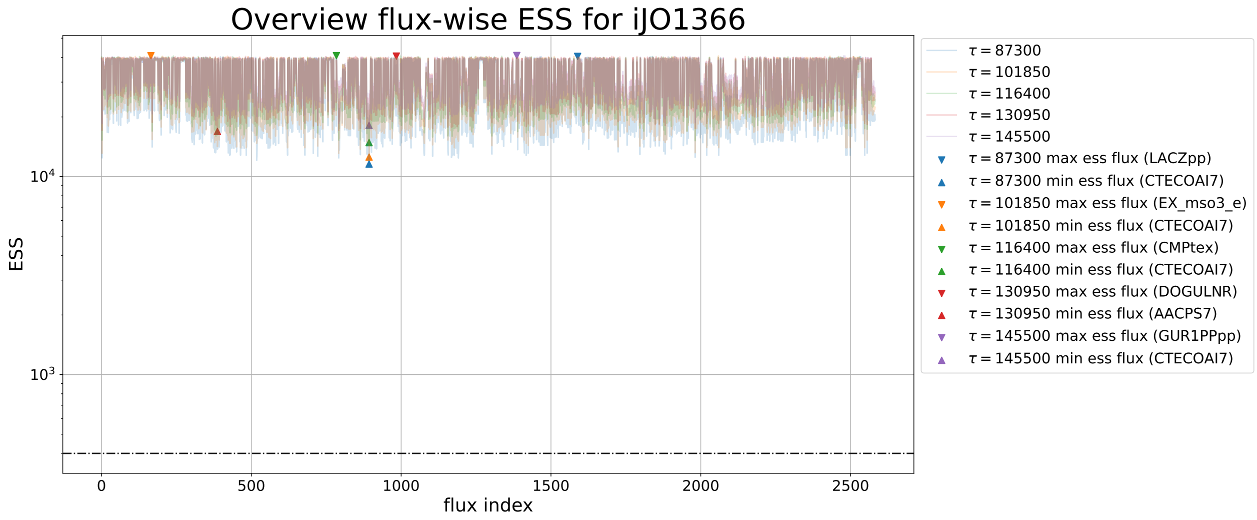

Figure E: **Flux-specific *ESS* for the genome-scale *iJO1366 E. coli* model for different thinning constants τ.** We see that the ESS values move closer together with increasing thinning constant. In this case, this equalizing of the ESS is not as visible because all displayed thinning constants τ are far from unthinned.

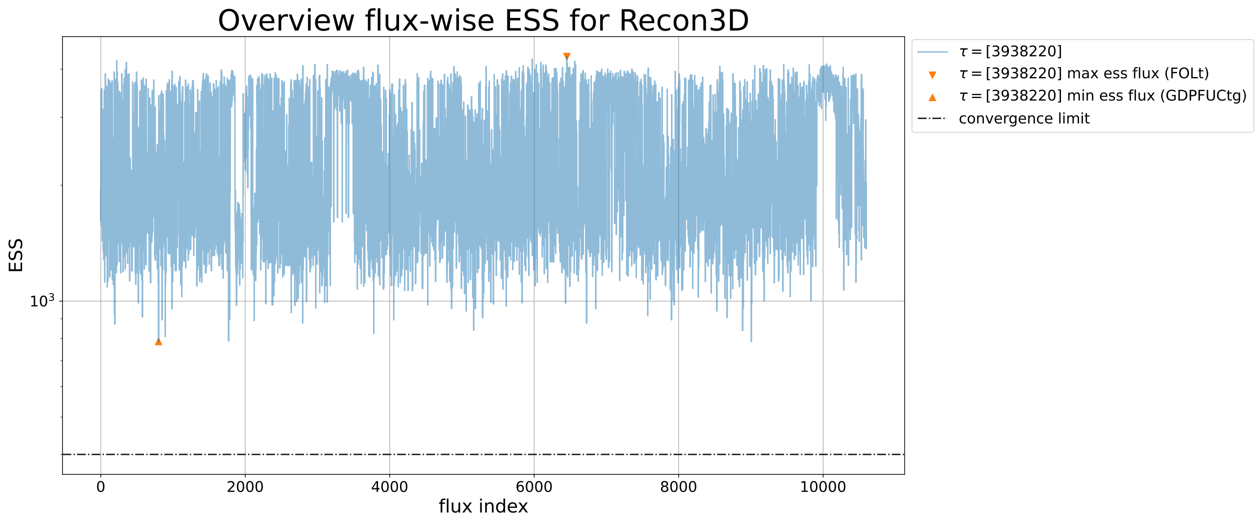

Figure F: **Flux-specific** ***ESS*** **for the** ***Recon3D*** **model for $\tau = d^2/6$, as recommended by our thinning guideline**. Fluxes do not mix equally well. However, there is no visible outlier, in the sense that no single flux mixes substantially better or worse than any other flux.

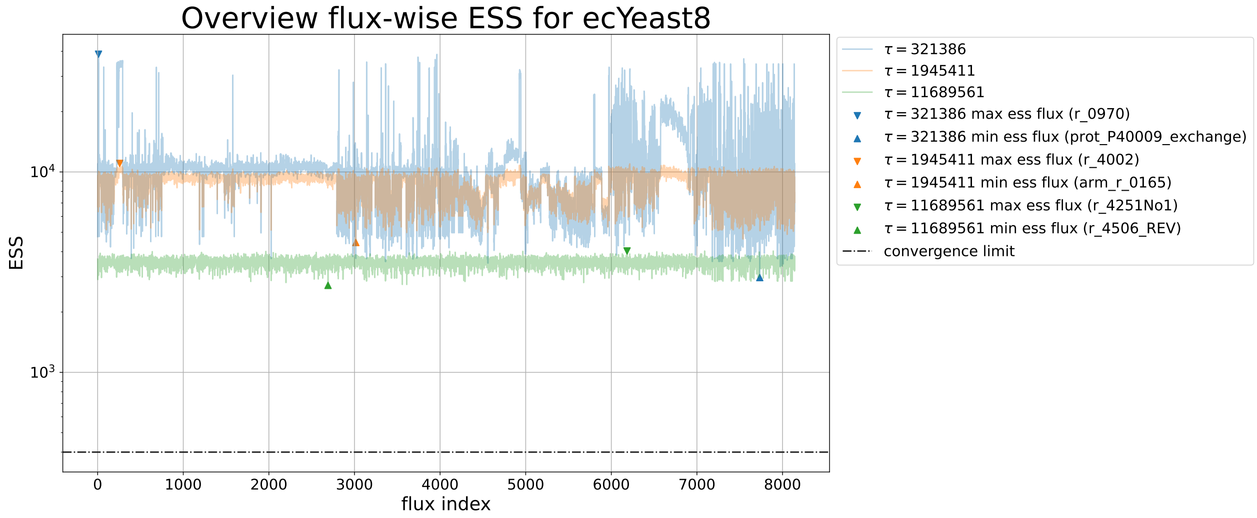

Figure G: **Flux-specific** ***ESS*** **for** ***ecYeast8*** **for different thinning constants τ.** As the thinning constant increases, the *ESS* is equalized.

6 Selected flux distributions

We here show exemplary pair-plots for selected fluxes from different pathways. Further pair-plots for all benchmarked models are available in our code-repository (https://jugit.fz-juelich.de/IBG-1/ModSim/Fluxomics/chrrt). Additionally, the selection of fluxes to include in the pair-plot can be altered in the jupyter-notebook.

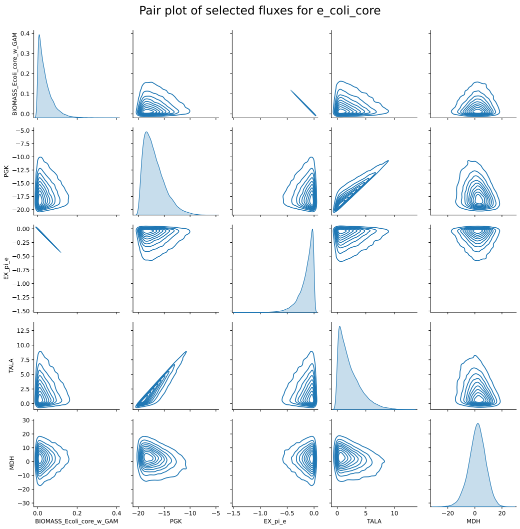

Figure H: **Selection of flux-distributions for the *e_coli_core* model.** The biomass flux strongly correlates with the phosphate exchange flux *EX_pi_e*.

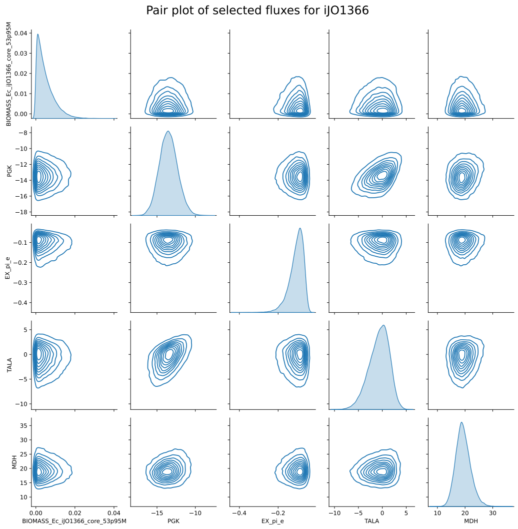

Figure I: **Selection of flux-distributions for the *iJO1366* model.** In contrast to the results with the core model in Fig H, the biomass flux is not strongly correlated with the phosphate exchange *EX_pi_e* in this *E. coli* model.

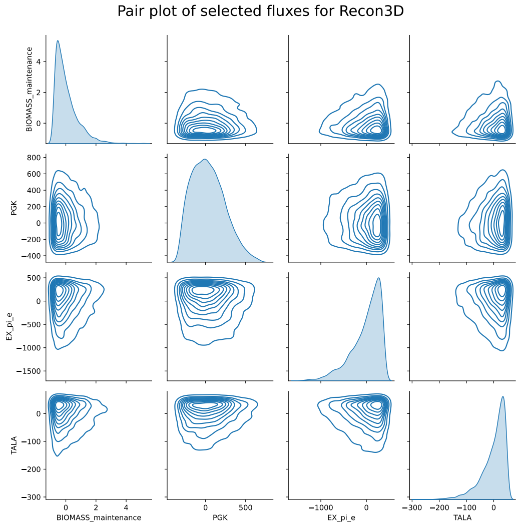

Figure J: **Selection of flux-distributions for the *Recon3D* model.**

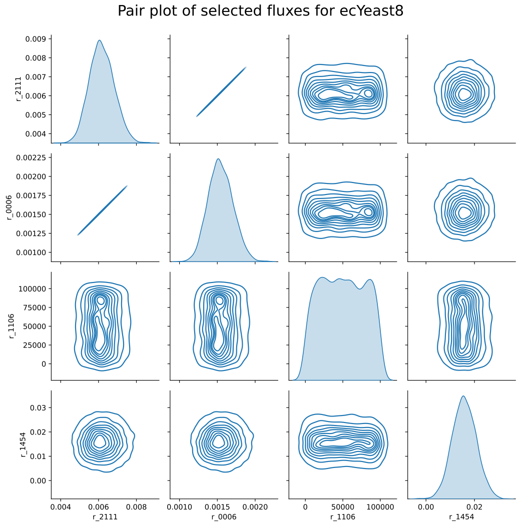

Figure K: **Selection of flux-distributions *ecYeast8*.**

Reference

[1] Axel Theorell, Johann F Jadebeck, Katharina Nöh, and Jörg Stelling. PolyRound: polytope rounding for random sampling in metabolic networks. *Bioinformatics*, 38(2):566–567, 2021. ISSN 1367-4803. doi: 10.1093/bioinformatics/btab552. URL `https://doi.org/10.1093/bioinformatics/btab552`.

[2] Jeffrey D. Orth, R. M. T. Fleming, Bernhard Ø. Palsson, and Peter D. Karp. Reconstruction and use of microbial metabolic networks: the core *Escherichia coli* metabolic model as an educational guide. *EcoSal Plus*, 4(1), 2010. doi: 10.1128/ecosalplus.10.2.1. URL `https://journals.asm.org/doi/abs/10.1128/ecosalplus.10.2.1`.

[3] Isabel S Shiratsubaki, Xin Fang, Rodolpho O O Souza, Bernhard O Palsson, Ariel M Silber, and Jair L Siqueira-Neto. Genome-scale metabolic models highlight stage-specific differences in essential metabolic pathways in *Trypanosoma cruzi*. *PLOS Neglected Tropical Diseases*, 14(10): e0008728, 2020. URL `https://doi.org/10.1371/journal.pntd.0008728`.

[4] Aarash Bordbar, Neema Jamshidi, and Bernhard O Palsson. *iAB-RBC-283*: A proteomically derived knowledge-base of erythrocyte metabolism that can be used to simulate its physiological and patho-physiological states. *BMC Systems Biology*, 5:110, 2011. URL `https://doi.org/10.1186/1752-0509-5-110`.

[5] Natalie C Duarte, Markus J Herrgård, and Bernhard Ø Palsson. Reconstruction and validation of *Saccharomyces cerevisiae* iND750, a fully compartmentalized genome-scale metabolic model. *Genome Research*, 14(7):1298–1309, 2004. URL `https://doi.org/10.1101/gr.2250904`.

[6] Alex Thomas, Sorena Rahmanian, Aarash Bordbar, Bernhard Ø Palsson, and Neema Jamshidi. Network reconstruction of platelet metabolism identifies metabolic signature for aspirin resistance. *Scientific Reports*, 4:3925, 2014. URL `https://doi.org/10.1038/srep03925`.

[7] Jeffrey D Orth, Tom M Conrad, Jessica Na, Joshua A Lerman, Hojung Nam, Adam M Feist, and Bernhard Ø Palsson. A comprehensive genome-scale reconstruction of *Escherichia coli* metabolism–2011. *Molecular Systems Biology*, 7:535, 2011. URL `https://doi.org/10.1038/msb.2011.65`.

[8] Yara Seif, Erol Kavvas, Jean-Christophe Lachance, James T Yurkovich, Sean-Paul Nuccio, Xin Fang, Edward Catoiu, Manuela Raffatellu, Bernhard O Palsson, and Jonathan M Monk. Genome-scale metabolic reconstructions of multiple Salmonella strains reveal serovar-specific metabolic traits. *Nature Communications*, 9(1):3771, 2018. URL `https://doi.org/10.1038/s41467-018-06112-5`.

[9] Natalie C Duarte, Scott A Becker, Neema Jamshidi, Ines Thiele, Monica L Mo, Thuy D Vo, Rohith Srivas, and Bernhard Ø Palsson. Global reconstruction of the human metabolic network based on genomic and bibliomic data. *Proceedings of the National Academy of Sciences of the United States of America*, 104(6):1777–1782, 2007. URL `https://doi.org/10.1073/pnas.0610772104`.

[10] Hongzhong Lu, Feiran Li, Benjamín J. Sánchez, Zhengming Zhu, Gang Li, Iván Domenzain, Simonas Marcišauskas, Petre Mihail Anton, Dimitra Lappa, Christian Lieven, Moritz Emanuel Beber, Nikolaus Sonnenschein, Eduard J. Kerkhoven, and Jens Nielsen. A consensus *S. cerevisiae* metabolic model yeast8 and its ecosystem for comprehensively probing cellular metabolism. *Nature Communications*, 10(1):3586, 2019. ISSN 2041-1723. doi: 10.1038/s41467-019-11581-3. URL `https://doi.org/10.1038/s41467-019-11581-3`.

[11] Elizabeth Brunk, Swagatika Sahoo, Daniel C. Zielinski, Ali Altunkaya, Andreas Dräger, Nathan Mih, Francesco Gatto, Avlant Nilsson, German Andres Preciat Gonzalez, Maike Kathrin Aurich, Andreas Prlić, Anand Sastry, Anna D. Danielsdottir, Almut Heinken, Alberto Noronha, Peter W. Rose, Stephen K. Burley, Ronan M. T. Fleming, Jens Nielsen, Ines Thiele, and Bernhard O. Palsson. *Recon3D* enables a three-dimensional view of gene variation in human metabolism. *Nature Biotechnology*, 36(3):272–281, 2018. ISSN 1546-1696. doi: 10.1038/nbt.4072. URL `https://doi.org/10.1038/nbt.4072`.

[12] Aki Vehtari, Andrew Gelman, Daniel Simpson, Bob Carpenter, and Paul-Christian Bürkner. Rank-normalization, folding, and localization: An improved $\widehat{R}$ for assessing convergence of MCMC (with discussion). *Bayesian Analysis*, 16(2):667 – 718, 2021. doi: 10.1214/20-BA1221. URL `https://doi.org/10.1214/20-BA1221`.
